# 3.B. Workshop: Quality of COVID-19 science: meta-research and the ethical implications for public health

**DOI:** 10.1093/eurpub/ckac129.125

**Published:** 2022-10-25

**Authors:** Organised by: EUPHA-EPI, EUPHA Research Pillar, EUPHA-ETH, EUPHA-PHG, EUPHA-HTA, UK Faculty of Public Health, Chair persons: Stefania Boccia (EUPHA-EPI), Alastair Leyland (EUPHA)

## Abstract

Since the beginning of the COVID-19 pandemic, scientific researchers have had to balance the need to disseminate information rapidly while still maintaining high-quality standards of research design and reporting. Early meta-research efforts comparing the methodological quality of COVID-19 research with pre-pandemic medical research found a higher prevalence of observational studies with a shorter time from submission to acceptance, lower methodological quality scores, higher risk of bias, and lower number of participants. This workshop is intended to showcase some public health relevant meta-research that has assessed COVID-19 study quality during later phases of the COVID-19 pandemic, analysed transparency and integrity indicators, and revisited earlier studies in the light of stronger evidence. After three more technical presentations that will examine the role of meta-research in addressing some of the COVID-19 pandemic research challenges, an ethical overview of the concept of “the science” as an idea used in public decision-making and policy will be given.

**Key messages:**

• Meta-research role of scrutinizing evidence as it is produced is even more important when scientific information is diffused at an expedited rate.

• Ethical questions might be as relevant as epidemiological ones when science is not able to be all that leads decision-making.

